# Impairment of microcirculation and vascular responsiveness in adolescents with primary Raynaud phenomenon

**DOI:** 10.1186/s12969-018-0237-x

**Published:** 2018-03-23

**Authors:** Bernadett Mosdósi, Kata Bölcskei, Zsuzsanna Helyes

**Affiliations:** 1 Clinical Center, Department of Pediatrics, University of Pécs, József Attila u. 7, Pécs, H-7623 Hungary; 2 János Szentágothai Research Centre & Centre for Neuroscience, University of Pécs, Ifjúság útja 20, Pécs, H-7624 Hungary; 3 Medical School, Department of Pharmacology and Pharmacotherapy, University of Pécs, Szigeti út 12, Pécs, H-7624 Hungary

**Keywords:** Primary Raynaud phenomenon, Adolescents, Microcirculation, Laser Doppler, Local hyperaemia

## Abstract

**Background:**

Raynaud’s phenomenon (RP) is a functional vascular disease, presenting with recurrent episodes of ischemia of extremities in response to cold and emotional stress. Investigating cutaneous microcirculation is an important tool in understanding the complex neuro-immuno-vascular interactions in its pathophysiological mechanisms. Since there is no available data on vascular responsiveness in RP in the paediatric population, we investigated skin perfusion and heat-induced hyperaemia in comparison with clinical severity and laboratory parameters of the disease.

**Methods:**

Fifty two adolescents (27 patients with primary RP and 25 age-matched healthy controls) were investigated in the study. Patients were divided into two groups according to the symptoms existing within the previous 2 months. Following baseline microcirculation measurement with Laser Doppler flowmetry (Periflux 5000 system), all subjects underwent local heating test at 42 °C and 44 °C. Besides routine laboratory parameters, immune-serological tests and the vasoactive sensory neuropeptides somatostatin and pituitary adenylate-cyclase activating polypeptide (PACAP) were measured.

**Results:**

Baseline perfusion measured in perfusion units (PU) at 32 °C was significantly lower in symptomatic RP patients (97.6 ± 22.4 PU) compared with both healthy volunteers (248.3 ± 23.5 PU, *p* < 0.001) and RP patients without symptoms (187.4 ± 24.9 PU, *p* < 0.05). After local heating to 42 °C maximum blood flow was significantly reduced in primary RP participants with current symptoms (358.6 ± 43.9 PU, *p* < 0.001), but not in asymptomatic ones (482.3 ± 28.7 PU, *p* > 0.05) when compared with healthy subjects (555.9 ± 28.2 PU). Both the area under the response curve and the latency to reach the maximum flow were significantly increased in both RP groups (symptomatic 164.6 ± 7.4 s, *p* < 0.001, asymptomatic 236.4 ± 17.4 s, p < 0.001) when compared with the control group (101.9 ± 4.7 s). The heat-induced percentage increase from baseline to maximal blood flow was significantly greater in symptomatic RP adolescents in comparison with healthy ones. Laboratory parameters and neuropeptide plasma levels were not altered in any groups.

**Conclusion:**

To our knowledge this is the first study in paediatric population to show altered heat-induced cutaneous hyperaemia responses in relation with the clinical severity and symptomatology.

## Background

Raynaud phenomenon (RP), first described by Maurice Raynaud in 1862 [1], is defined as recurrent, reversible episodes of vasospasm involving peripheral small vessels. The fingers are the most commonly affected regions and RP is typically triggered by cold exposure, emotional stress and exercise [2]. The phenomenon is manifested clinically by sharply demarcated colour changes of the skin of the digits, and can be classified as primary and secondary. Primary RP accounting for 80% of the cases is present without any associated diseases explaining the symptoms [3]. Secondary RP is associated with other conditions, mainly connective tissue diseases (CTD), endocrine and hyperviscosity disorders, as well as drug exposure. It commonly occurs (80–90%) in children with systemic sclerosis (SSc) and CTD. The differentiation between the two forms is important, because primary RP has good outcome, but careful monitoring is essential to ensure early detection and management of evolving CTD [4–6].

The prevalence of RP is more common among women and family members of RP patients [7], it is approximately 3–20% in the total population [2, 3, 8]. However, the pediatric prevalence is not well known. A study of 720 school-children at the age of 12–15 years in the UK reported a prevalence of 18% in females and 12% of males [9].

Cutaneous blood flow is regulated by complex neuro-immune-humoral mechanisms, involving both the autonomic and sensory nervous systems. Mediators are hormones and vasoactive compounds released from nerves, circulating cells and blood vessels. The pathophysiology of RP is poorly understood, but the key mechanism is related to the imbalance between vasoconstrictor and vasodilator events. A complex disorder of several neuroendocrine interactions and local production of reactive oxygen species resulting in decreased nitric oxide (NO) production lead to intensified vasoconstriction [8, 10–13]. Sensory nerves play a predominant role in the regulation of vascular responses elicited by thermal stimuli. Peptide mediators released from autonomic or sensory nerve endings, such as calcitonin gene related peptide (CGRP) [14], vasoactive intestinal peptide (VIP) or the closely related pituitary adenylate cyclase activating polypeptide (PACAP) [15, 16] dilate the vessels by directly relaxing the vascular smooth muscle and indirectly via the endothelial release of NO [12]. Indeed, reduction of CGRP- and VIP-positive nerves in the skin of adult RP patients has been described [17, 18]. On the other hand, it is well known that secondary RP in SSc is also caused by structural microvascular changes, such as endothelial dysfunction and fibrous intimal proliferation with associated intravascular thrombi [19].

In order to get closer to the pathophysiological mechanisms, it is important to investigate cutaneous microvascular function in RP. Skin microcirculation can be examined by both non-invasive [20–22] and invasive techniques, such as biopsies [17] or intradermal delivery of drugs [23]. Laser Doppler flowmetry is a sensitive, non-invasive method for the measurement of tissue perfusion. The monochromatic laser beam penetrates the skin, it is reflected depending on the movement velocity of the red blood cells and recorded by a sensitive sensor. Cold stimulation-induced skin blood flow responses were found to be unaltered in adult RP and SSc patients, as compared to healthy subjects [24]. Meanwhile, several other studies in adults showed a dramatic alteration of the amplitude and the kinetics of post-occlusive hyperaemia in patient with SSc compared to primary RP and healthy controls [25, 26]. However, thermal hyperaemia was more sensitive and specific than post-occlusive hyperaemia for differentiating SSc from primary RP [27]. All these data clearly show that investigating vascular responsiveness in the skin, with special emphasis on heat-induced vasodilatation is a valuable tool to understand the pathophysiological background of RP, but there are no data in paediatric population.

Therefore, the aim of the present study was to investigate cutaneous microcirculatory alterations in RP adolescents and analyse the heat-induced microvascular responsiveness in relation to the clinical symptoms.

## Methods

### Patient selection and ethics

Fifty-two adolescents were enrolled in this study at the Department of Pediatrics of the Clinical Centre of the University of Pécs, Hungary in March and April of 2015.

Primary RP was diagnosed according to the criteria of LeRoy [28], including a normal nail-fold capillaroscopy (characterized by homogeneous distribution of capillary loops similar in shape and size), the lack of antinuclear antibodies, no digital pitting scar and the lack of clinical symptoms of CTD. Exclusion criteria were cigarette smoking, chronic diseases such as CTD, diabetes mellitus, hypercholesterolemia, hypertonia and any drug therapy. Two eligible RP participants were later also excluded from the study due to difficulties with the microcirculation measurement analysis.

Healthy adolescents were recruited through letters sent to the local secondary schools. Overall, there were 27 patients with primary Raynaud syndrome and 25 age- and gender-matched healthy controls.

The study was performed according to the recommendations of the Declaration of Helsinki and the protocol was approved by the Local Ethics Committee (5501/2015). Informed consent was obtained from all adolescents and their parents.

### Measurement protocol and paradigm

On the day of the investigation, the participants arrived at the department between 7:30 a.m. and 9 a.m. in the fasting state. Measurements were performed within one day in a quiet room at 23.0 ± 0.5 °C temperature. After a thorough physical examination, body composition analysis was done with Tanita BC 420 MA Body Composition Analyser [29]. Afterwards, subjects were placed in a supine position with both forearms resting at heart level. Five minutes later, heart rate and blood pressure were measured.

Venous blood samples were taken for routine laboratory tests including haematocrit (Htc), haemoglobin (Hgb), white blood cell count (WBC), platelet count, differential blood smear, erythrocyte sedimentation rate (ESR), C- reactive protein (CRP), liver and kidney function, thyroid stimulating hormone (TSH), coagulation tests, immunoglobulin and complements (C3,C4). Immunoserological tests included the measurements of antinuclear (ANA), anti-dsDNA, anti-centromere (ACA), anti-C1Q, anti-extractable nuclear antibody (ENA), anticardiolipin and anti-beta 2 glycoprotein, antiprothrombin antibody levels. Routine urine analysis was also performed. Blood samples were also collected for measuring vasoactive sensory neuropeptides, such as PACAP-38 and somatostatin by specific and sensitive radioimmunoassay (RIA) techniques developed in our laboratory [30, 31]. Peptidase inhibitor aprotinin (200 IU per ml of blood; Gordox, Gedeon Richter, Budapest) was added immediately to the blood taken into EDTA tubes. Plasma was obtained by centrifugation of whole blood at 4 °C degree 1000 g for 4 min and then 4000 g for 15 min and aliquots were stored at −70 °C.

**Fig. 1:**
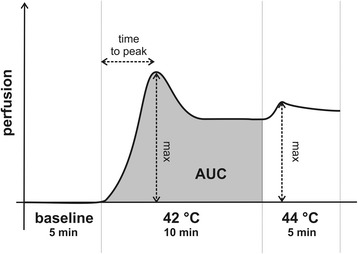
Cutaneous microcirculation changes and evaluated parameters on the left index finger in response to heating (area under the response curve: AUC)

The participants were resting comfortably for 5 min and their arms were immobilised to ensure stable positioning. Microcirculation on the left index finger was measured with the Periflux 5000 system (Perimed AB, Stockholm, Sweden) Laser Doppler technology. A thermostatic Laser Doppler probe (Probe 457) was placed on the distal phalanx of the second finger of the left hand. Data from the Laser Doppler flowmeter were interfaced to a computer, the perfusion measured in arbitrary perfusion units (PU) and the kinetics of the response was determined by the time. Baseline mean temperature was maintained at 32 °C and blood flow recorded over 5 min. The laser probe was heated to 42 °C for 10 min and then 44 °C for a period of maximum 5 min. Microcirculation parameters, such as heat-induced hyperaemia were expressed as the area under the response curve (AUC), time to peak response (the time to attain maximal cutaneous perfusion in seconds), peak perfusion value and percentage increase (Fig. 1). The cooling test could not be performed because of the symptoms of RP patients.

### Data analysis and statistics

Data management and analysis were performed using GraphPad Prism version 5.0 (GraphPad Software, San Diego, CA). Values were expressed as means ± SEM in all the 3 groups of subjects. The normal distribution of investigated parameters was confirmed by D’Agostino-Pearson and Shapiro-Wilk normality tests. In case of normal distribution, values for each group were compared by one-way analysis of variance (ANOVA) followed by Newman-Keuls multiple comparison test. Otherwise, the Kruskal-Wallis test followed by Dunn’s multiple comparison test was used for statistical analysis. A value of *p* < 0.05 was considered as statistically significant. Correlation coefficients between selected clinical and microcirculation parameters were also analysed.

## Results

### Clinical and laboratory characteristics of the patients

The demographics and clinical characteristics of the 52 adolescents investigated in the study are listed in Table 1. Patients with Raynaud symptoms were divided into two groups according to the symptoms existing on the day of the study or in the past 2 months. Four of the participants included in the control group were diagnosed based on medical history and physical examination with primary RP and were therefore switched to the group of Raynaud patients without current symptoms. Only one of the patients without current symptoms of RP had Tanner stage III, all the rest had Tanner stage IV.

**Table 1:** Demographic and clinical characteristics of adolescents involved in the study

	Healthy	Raynaud without current symptoms	Raynaud with current symptoms
Number	25	15	12
Age (years)	16.5 ± 0.2 (14.8–19)	16.6 ± 0.4 (13.2–18.2)	16.2 ± 0.4 (13–18)
Duration of the disease (months)	–	23.2 ± 4.9 (2.4–54)	38.4 ± 6.9 (12–96)
Body fat composition (%)	24.0 ± 1.5 (4.3–41.8)	32.1 ± 2.7 (17.8–50.6)**	18.1 ± 1.7 (8.2–27.4)*, ^###^
Body Mass Index (BMI)	22.0 ± 0.8 (17.3–35.1)	25.8 ± 1.9 (17.6–39.5)	18.78 ± 0.4 (15.8–20.8) *, ^##^
Mean arterial blood pressure (mmHg)	87.5 ± 1.7 (73.3–107.0)	90.1 ± 1.6 (82–101.3)	81.7 ± 2.1 (71.7–97.0)*, ^#^

Data represent the means±SEM, as well as the range of the data in each group (*p < 0.05, ** *p* < 0.01 vs. healthy; ^#^*p* < 0.05, ^##^p<0.01, ^###^*p* < 0.001 vs. patients without current symptoms, Kruskal-Wallis test followed by Dunn’s multiple comparison test for body mass index values, one-way ANOVA followed by Neuman-Keuls multiple comparison test for all other parameters)

Most patients except one in each group among the Raynaud patients were girls. The mean duration of primary RP was 23.2 ± 4.9 months in the group of Raynaud without symptoms and 38.4 ± 6.9 months in the group of Raynaud with symptoms, the difference, however, was not statistically significant. Compared to healthy controls body fat percentage values were significantly higher in Raynaud children without symptoms and significantly lower in symptomatic patients compared to controls. BMI was also significantly lower in symptomatic patients compared to either healthy controls or patients with symptoms. In the group of RP with symptoms 1 person was found to be severely underweight, and 3 were underweight but none of the patients were anorectic. At the same time in the group of RP without symptoms only one patient was underweight, however four of them were obese. The difference in BMI was highly significant between the two patient groups, as well.

The mean arterial blood pressure was also lower in Raynaud patients with symptoms compared with either healthy or asymptomatic patients (Table 1). Participants were asked to rate their symptoms based on numbness, pain, and the frequency and duration of attacks. Patient-reported subjective symptom severity was moderate to severe. Ischemic ulcerations were not observed in any cases at the time of the study. The history and clinical examination did not show any signs of CTD. The laboratory parameters were in the normal range in all subjects. None of them had positive autoantibodies against nuclear (ANA), topoisomerase I (SCL-70) or centromere-associated (ACA) proteins.

Nail-fold capillaroscopy did not show scleroderma pattern characterized by enlarged capillaries, giant capillaries, haemorrhages, or loss of capillaries, disorganization of capillaries architecture in any of the subjects. We only found non-specific patterns, minor capillary morphological changes (mean disarrangement of capillary density and capillary polarity, nonhomogeneous distribution or size of loops, linear elongation of the loop) which can even be found in primary RP patients and they are not considered pathological.

### Disease activity-relation of microcirculatory impairment in adolescents with Raynaud phenomenon

**Fig. 2:**
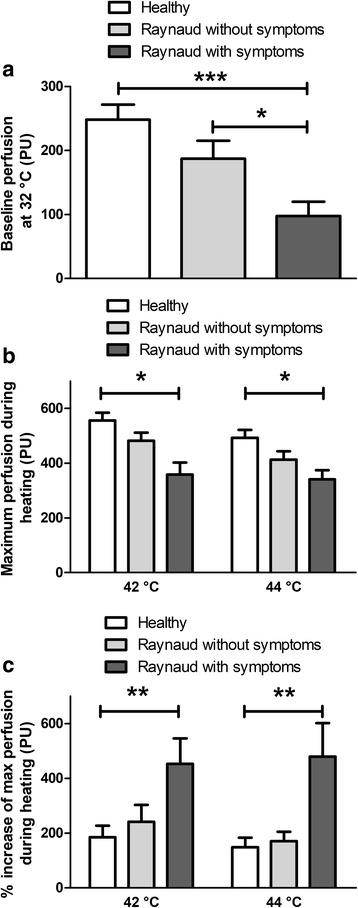
Baseline perfusion (**a**) demonstrated as perfusion units (PU), maximum perfusion in response to heating to 42 °C and 44 °C (**b**), percentage perfusion increase during heating (**c**). Columns represent the means±SEM of each group (*p < 0.05, ** *p* < 0.01, ***p < 0.001; one-way ANOVA followed by Neuman-Keuls multiple comparison test)

Baseline perfusion at 32 °C was 97.6 ± 22.4 perfusion units (PU) in symptomatic Raynaud patients which was significantly lower compared with either healthy volunteers (248.3 ± 23.5 PU, *p* < 0.001) or patients without symptoms (187.4 ± 27.9 PU, *p* < 0.05). The difference between healthy controls and asymptomatic participants was not statistically significant. (Fig. 2a). Heating to 42 °C and 44 °C induced a gradual increase in cutaneous blood flow. The maximum blood flow at 42 °C was significantly reduced in RP adolescents with current symptoms (358.6 ± 43.9 PU), compared with healthy subjects (555.9 ± 8.2 PU, *p* < 0.05). However, there was no significant difference in this response parameter between healthy controls and patients without symptoms (482.3 ± 28.7 PU) or between the two Raynaud groups (Fig. 2b). Analysing the percentage changes from baseline to maximal flow during heating to 42 °C, significantly greater increase was detected in symptomatic RP adolescents (452.9 ± 93.4%) compared with either the controls (185.1 ± 42.5%, *p* < 0.01), or the asymptomatic RP patients (241.7 ± 61.5%, *p* < 0.05). This parameter did not differ in the disease group without symptoms in comparison with healthy subjects, similarly to the absolute perfusion values (Fig. 2c). Heating to 44 °C induced perfusion changes similar to the 42 °C stimulus and statistical comparisons revealed the same differences between groups (Fig. 2b and c).

**Fig. 3:**
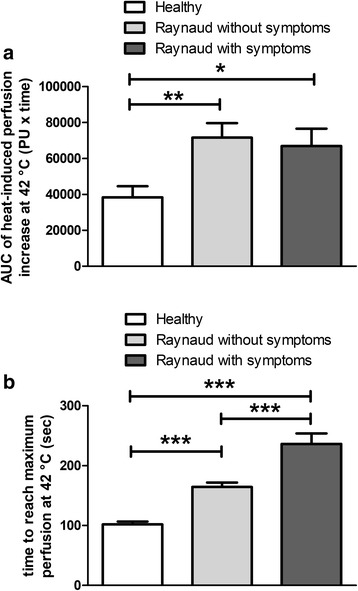
Heat-induced hyperaemia response (**a**), and (**b**) latency to reach the maximal response. Columns represent the means±SEM of each group (*p < 0.05, ** *p* < 0.01, ***p < 0.001; one-way ANOVA followed by Neuman-Keuls multiple comparison test)

Additional analysis of the perfusion change revealed that the kinetics of the heat-induced response was also altered in patients with RP in comparison with healthy controls. The AUC of the 42 °C heat-induced perfusion response was significantly greater in both Raynaud disease groups compared with the control group. No significant difference in AUC was found between symptomatic and asymptomatic patients (Fig. 3a**)**.

On the other hand, latency to reach the maximum perfusion at 42 °C was significantly longer in both patient groups (symptomatic: 236.4 ± 17.4 s, asymptomatic 164.6 ± 7.4 s) compared with healthy controls (101.9 ± 4.7 s, *p* < 0.001 for both). Moreover, the latency was also significantly different between the two RP patient groups (*p* < 0.001, Fig. 3b).

Correlations between microcirculatory parameters and mean blood pressure or body fat percentage or disease duration were analysed, but no significant relationships were detected (data not shown).

Plasma concentrations of vasoactive sensory neuropeptides were not altered in primary Raynaud adolescents. PACAP-38 and somatostatin-LI were reliably measurable in the systemic circulation, however there were no significant differences between their concentrations in the three groups (Table 2.).

**Table 2:** Plasma somatostatin (SOM) and pituitary adenylate cyclase activating polypeptide-38 (PACAP-38) levels determined by radioimmunoassay in healthy controls and Raynaud patients with or without symptoms. Data represent the means±SEM of each group

	Healthy	Raynaud without symptoms	Raynaud with symptoms
Somatostatin (fmol/ml)	4.9 ± 0.3	4.9 ± 0.4	5.6 ± 0.5
PACAP-38 (fmol/ml)	15.8 ± 1.6	15.2 ± 1.5	13.1 ± 1.4

## Discussion

To our knowledge this is the first study in a paediatric primary RP population to show altered heat-induced cutaneous hyperaemia responses in relation with the clinical severity and symptomatology.

Detection of tissue perfusion dysfunction is necessary for the early diagnosis of microvascular disease. Laser Doppler flowmetry has been proven to be a valuable non-invasive method for the diagnosis and follow-up of peripheral vascular diseases such as diabetic microangiopathy, atherosclerosis, and wound healing after burns or reconstructive surgery [32–35]. Since baseline perfusion values depend on a variety of exogenous and endogenous factors, the results of provocation tests (pressure, heat, cold or chemical stimulation) should be used for reliable comparison. Local heat-induced skin hyperaemia is based on two separate mechanisms: a sensory nerve-mediated initial peak and a sustained plateau phase dependent on endothelial factors, mostly NO [36, 37]. Previous studies in the adult primary RP population showed that the initial neurogenic vasodilator response to heating was not affected by the disease [27, 38]. Few capillary microscopy studies have investigated the vasculature in RP children, but none of them described functional investigations of responsiveness to heat [39, 40].

Similarly to previous adult studies we also found significantly decreased baseline blood flow in primary RP adolescents, but only in the symptomatic group [41, 42]. This can be partially explained by the higher body weight and fat percentage in the population of asymptomatic RP, since BMI has an impact on the regulation of skin temperature and perfusion. It has been demonstrated that weight could have an impact on sympathetic nerve–dependent regulation of vascular tone [43] and studies in obese children also reported increased skin perfusion [44, 45]. Furthermore, BMI was shown to be related to skin temperature and skin perfusion in adult patients with primary RP, but not in healthy controls [46].

We showed that both the kinetics and amplitude of the hyperaemic response to local heating were altered in RP in comparison with healthy participants. It should be emphasized that the kinetics of the thermal response could distinguish between symptomatic and asymptomatic RP children. Peak perfusion values during heating were significantly lower and responses were significantly delayed in symptomatic primary RP adolescents. These findings are fundamentally different from the results reported in adult primary RP patients [27]. Unexpectedly, the percentage increase above baseline, as well as the AUC values were significantly higher in the symptomatic group compared to healthy controls. It might suggest a functional reserve capacity of the microvasculature in these patients. The higher relative increase might be explained by a greater sensitivity of RP patients to intravenous CGRP, as described in adults [47], although this finding was not confirmed by others [48, 49]. In contrast, no difference was observed in the maximum perfusion or in the percentage increase between asymptomatic RP patients and controls. However, the AUC and latency to reach the maximum perfusion were significantly higher, demonstrating an overall difference between asymptomatic primary RP and healthy adolescents. The most sensitive parameter proved to be the latency value, since it could differentiate between the three study groups. In adults the response to local heating was described as a characteristic to distinguish secondary RP patients from primary ones [27], but to our knowledge, ours were the first to use Laser Doppler flowmetry as a tool to detect the disease severity in the paediatric RP population.

A complex interplay of several neuroendocrine mechanisms with local pathways is present in RP and CTD. The pathophysiology of primary RP is multifactorial with both vascular and neural abnormalities. The loss of CGRP- and VIP-containing nerves in the cutaneous microvasculature [17, 18] and abnormal response of cold-sensitive nerves were described in primary adult RP patients [50]. A recent study found a strong relationship between microangiopathy and higher serum endothelin-1 and E-selectin levels in children with RP [40], although previous data had not confirmed the diagnostic value of endothelin concentration [51]. It is well-established that peptide mediators play a role in neurovascular responses, but there is limited data about neuropeptide concentration changes in primary RP [12]. We found no plasma concentration alterations of vasoactive sensory neuropeptides in primary RP adolescents compared with healthy controls. The lack of systemic changes of neuropeptide levels suggests that only local neuroendocrine mechanism may be involved in a shift towards vasoconstriction.

Some potential limitations should be considered when interpreting our results. First, it was a monocentric study. The number of participants was small, but we strictly compared age and sex-matched groups to avoid possible bias in pubertal development. In order to minimize a possible bias by differences in physical examination and microcirculation measurement with Laser Doppler flowmetry, the same investigator did the procedure. A disadvantage of Laser Doppler flowmetry is the dependence of the signal of other variables such as skin thickness or temperature. Therefore, care was taken to perform perfusion measurements at the same site under standardized, identical conditions and a preheated probe was used to standardize baseline cutaneous temperature. Despite these limitations, assessment of perfusion changes by Laser Doppler flowmetry is considered accurate and reliable for diagnostic purposes of peripheral arterial disease [52, 53].

## Conclusion

To our knowledge, this is the first study in paediatric RP population to show altered heat-induced cutaneous hyperaemia responses in relation to the clinical severity and symptomatology using a sensitive, easy and non-invasive method. Since CTD develop in 3–40% of RP subjects [4–6], early detection is important for better treatment results and prognosis. Previous studies suggest that in CTD microcirculatory changes develop before morphological abnormalities are seen with nail-fold capillaroscopy [54–56]. Therefore, altered microvascular response to thermal stimuli could be an early marker, but a follow-up study is needed to determine whether this parameter could be an objective severity and prognosis indicator.

## Abbreviations

AUC: Area under the curve
CGRP: Calcitonin gene-related peptide
CTD: Connective tissue disease
NO: Nitric oxide
PACAP: Pituitary adenylate cyclase activating polypeptide
RP: Raynaud phenomenon
SOM: Somatostatin
SSc: Systemic sclerosis
